# Effect of an omega-3 lipid emulsion in reducing oxidative stress in a rat model of intestinal ischemia−reperfusion injury

**DOI:** 10.1007/s00383-012-3144-0

**Published:** 2012-08-21

**Authors:** Atsuhiro Arisue, Naoki Shimojima, Masayuki Tomiya, Takayuki Shimizu, Daisuke Harada, Mitsuo Nakayama, Hirofumi Tomita, Masahiro Shinoda, Minoru Tanabe, Ikuro Maruyama, Masaru Mizuno, Tatsuo Kuroda, Go Wakabayashi, Yasuhide Morikawa

**Affiliations:** 1 School of Medicine, Department of Pediatric Surgery, Keio University, 35 Shinanomachi, Shinjuku-ku, Tokyo, 160-8582 Japan; 2 School of Medicine, Department of Surgery, Iwate Medical University, Morioka, Japan; 3 Laboratory of Clinical Nutrition, Naruto Research Institute, Otsuka Pharmaceutical Factory, Inc, Naruto, Japan; 4 Department of Laboratory and Vascular Medicine, Kagoshima University Graduate School of Medicine and Dental Sciences, Kagoshima, Japan; 5 Pediatric Surgery, International University Medical Welfare Hospital, Nasushiobara, Japan

**Keywords:** Ischemia–reperfusion injury, Omega-3, Oxidative stress, HMGB1

## Abstract

**Objectives:**

The usefulness of omega-3 lipid emulsions has been extensively studied. The objectives of the present study were to examine the effect of an omega-3 lipid emulsion in reducing oxidative stress in a rat model of intestinal ischemia−reperfusion injury and the underlying mechanism.

**Methods:**

A total of 66 rats were divided into three dietary groups (lipid-free, soybean oil, and fish oil groups). Each animal was administered total parenteral nutrition for 3 days, followed by induction of intestinal ischemia for 100 min. Animals subjected to sham surgery served as the controls. Intestinal tissue and blood were harvested 6 and 12 h after the surgery, then, assessment of the histological damage score, plasma-related parameters, and statistical evaluation were performed.

**Results:**

The histological damage score in the intestinal tissues was significantly lower in the fish oil group than in the soybean oil group (*P* = 0.0121). The late-phase urinary level of 8-hydroxy-2-deoxyguanosine was also significantly lower in the fish oil group as compared with that in the other groups (*P* = 0.0267). Furthermore, the plasma level of high-mobility group box 1 protein was also significantly lower in the fish oil group as compared with that in the lipid-free group (*P* = 0.0398).

**Conclusion:**

It appeared that intravenous administration of an omega-3 lipid emulsion prior to ischemia−reperfusion injury reduced the oxidative stress and severity of tissue damage. Modification of membrane fatty acids may serve as the mechanism underlying this reduction of tissue damage.

## Introduction

In recent years, the beneficial effects of omega-3 lipid emulsions have been extensively studied in various pathological conditions, including in models of pancreatitis [1], ischemia−reperfusion injury of the small intestine [2, 3], and nonalcoholic steatohepatitis (NASH) [4]. On the other hand, in the clinical setting, acute and chronic administration of general-lipid-strengthened parenteral nutrition has been reported to be associated with elevated risk of liver disorders [5], coronary artery disease, inflammatory bowel disease, and NASH [6].

As an exception to the above-mentioned, omega-3 lipid emulsions have been suggested to reduce the extent of tissue damage in intestinal ischemia−reperfusion injury by decreasing the oxidative stress. Although this protective effect has been observed in previous studies using rat models of ischemia−reperfusion injury of the small intestine [2, 3], the pathogenesis of the injury and the mechanism underlying the protective effect of omega-3 lipid emulsions against this injury remain poorly understood. The aim of the present study was to evaluate the protective effect of omega-3 lipid emulsions against tissue damage in intestinal ischemia−reperfusion injury, which is commonly seen in ischemic bowel disease, ischemia−reperfusion injury, and small bowel transplantation.

## Materials and methods

### Experimental animals

Male Crlj: WI rats (250−300 g, 7−8 weeks) obtained from Charles River Laboratories (Yokohama, Japan) were housed in aluminum cages at room temperature (23 ± 3 °C, humidity of 55 ± 3 *%*) under a 12-h light–dark cycle. All procedures were approved by the Keio University Animal Ethics Committee and Committee on the Animal Experiments of Otsuka Pharmaceutical Factory, Inc.

### Experimental design

One week prior to the start of the experiment, the rats were fed a modified AIN-93G diet (Nosan Corporation, Yokohama, Japan), containing a soybean-oil-derived lipid and no fish oil. A central venous catheter (advanced silicon-body plastic tube, 0.5−1.0 mm) was inserted into the internal jugular vein of each rat after the animal had been denied access to food for 12 h, while water was still made available ad libitum. Total parenteral nutrition (TPN) was started on day 0. The rats were divided into three groups (fish oil, soybean oil, and lipid-free groups), and were administered different components of lipids, as shown in Table 1. Omegaven (Fresenius Kabi GmbH, Linz, Austria) was used as the fish-oil-enriched lipid emulsion, and Intralipos Injection 10 % (Otsuka Pharmacy, Naruto, Japan) was used as the soybean-oil-enriched lipid emulsion. TPN was administered for 3 days based upon previous observation by the co-authors of membrane fatty acid changes after 3 days of TPN [7]. Each animal was given a standard caloric supply of 210 kcal/kg/day, corresponding to 30 kcal/kg/day in humans. In the lipid mixture group, 30 % of the total calories were derived from fat. An identical amount of calories was provided by carbohydrates in the lipid-free group. The ratio of amino acids:lipids:glucose of 13:30:57 during TPN was applied according to the recommendation of the European Society for Parenteral and Enteral Nutrition and Metabolism (ESPEN) [8]. On day 3, after a 2-h infusion of extracellular fluid, the rats were subjected to intestinal ischemia–reperfusion, as described below, or sham surgery. After the surgery, the infusion of extracellular fluid, and food and water were withheld. Rats were killed 6 h or 12 h after reperfusion, and intestinal tissue, urine, and blood samples were harvested.

**Table 1:** Components of infusion solutions

	TPN with lipid-free	TPN with soybean oil	TPN with fish oil
Water volume (mL/kg/day)	260	260	260
Glucose (g/kg/day)	46	30	30
Amino acids (g/kg/day)	7	7	7
Lipids (g/kg/day)	0	7	7
Total calories (kcal/kg/day)	211	211	211

A total of 66 rats were randomly assigned to the following six groups:

1. lipid-free TPN undergoing sham surgery, killed after 6 h (*n* = 3) and killed after 12 h (*n* = 5)
2. soybean oil TPN undergoing sham surgery, killed after 6 h (*n* = 3) and killed after 12 h (*n* = 5)
3. fish oil TPN undergoing sham surgery, killed after 6 h (*n* = 3) and killed after 12 h (*n* = 5)
4. lipid-free TPN undergoing reperfusion surgery, killed after 6 h (*n* = 6) and killed after 12 h (*n* = 8)
5. soybean oil TPN undergoing reperfusion surgery, killed after 6 h (*n* = 6) and killed after 12 h (*n* = 8)
6. fish oil TPN undergoing reperfusion surgery, killed after 6 h (*n* = 6) and killed after 12 h (*n* = 8)

### Surgical techniques

The experimental animals were handled as previously reported [9]. General anesthesia was administered by isoflurane inhalation. The superior mesenteric artery was occluded with a clamp, and the small bowels were reperfused after 100 min of ischemia.

### Histological assessment of the intestine

5 cm specimens of the small intestine were randomly harvested from a region 10 cm proximal to the terminal ileum and processed for histological examination; after the specimens were fixed in formaldehyde (10 %), they were stained with hematoxylin−eosin. Each intestinal specimen was scored for evaluating the severity of tissue damage using the Park injury scoring system. The scores in this rubric grade from 0 to 8 (Table 2; [10]). To reduce sampling error, each sample was divided into four parts, and each part was evaluated.

**Table 2:** The severity of tissue damage using the Park injury scoring system

Grading	Morphological change
0	Normal mucosa
1	Subepithelial Gruenhagen‘s space at villus tip
2	Extended subepithelial villus sides
3	Epithelial lifting along villus sides
4	Denuded villi
5	Loss of villus tissue
6	Crypt layer infarction
7	Transmucosal infarction
8	Transmural infarction

### Biomarkers

Urine samples were collected 0−6 h after the reperfusion (early-phase urine samples) and 6−12 h after reperfusion (late-phase urine samples) and preserved in a freezer at −80 °C. Blood samples were collected from the inferior vena cava soon after the animals were killed, and the plasma specimens were stored in a freezer at −80 °C. Plasma levels of oxidative stress markers levels, including 8-hydroxy-2-deoxyguanosine (8-OHdG) and isoprostane, and also the concentrations of prostaglandin E2 (PGE2), and high-mobility group box 1 (HMGB1) protein were measured by enzyme-linked immunosorbent assay (ELISA). (The HMGB1 ELISA kit of Shino-Test Corporation, and oxidative stress marker ELISA kit of Japan Institute for the Control of Aging, NIKKEN SEIL Co., Ltd., were used for this study.)

### Statistical analysis

Statistical significance was set at *P* = 0.05. In two-group comparisons, Bonferroni correction was used for adjustment of the significance level (*P* = 0.05/2). Statistical analysis was carried out using *EXSUS* (CAC Corporation), based on the *SAS (*SAS Institute Ltd.). Histopathological scores were statistically compared among the groups using Dunnett’s test,* F* test, student’s *t* test, and the Aspin−Welch test. Urine and blood sample scores are presented as mean ± SD. Dunnett’s tests were used for two-group comparisons (i.e., comparison between the lipid-free and soybean oil groups, lipid-free and fish oil groups, and, the soybean oil and fish oil groups).

## Results

### Histology of the small intestine

**Fig. 1:**
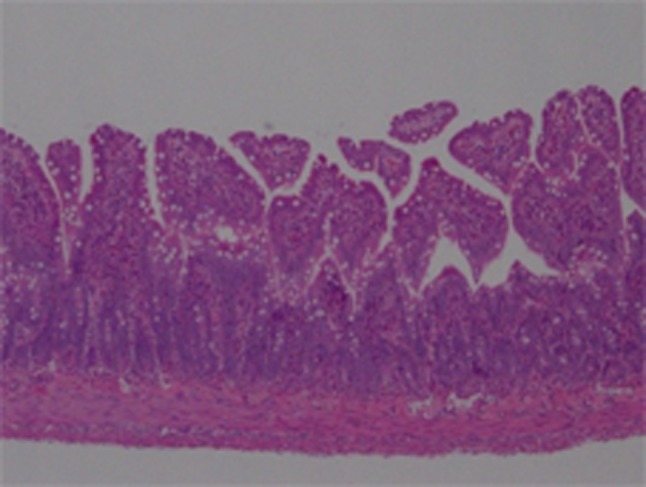
Histological findings in sham surgery group. No mucosal damage was seen (hematoxylin−eosin staining×100)

**Fig. 2:**
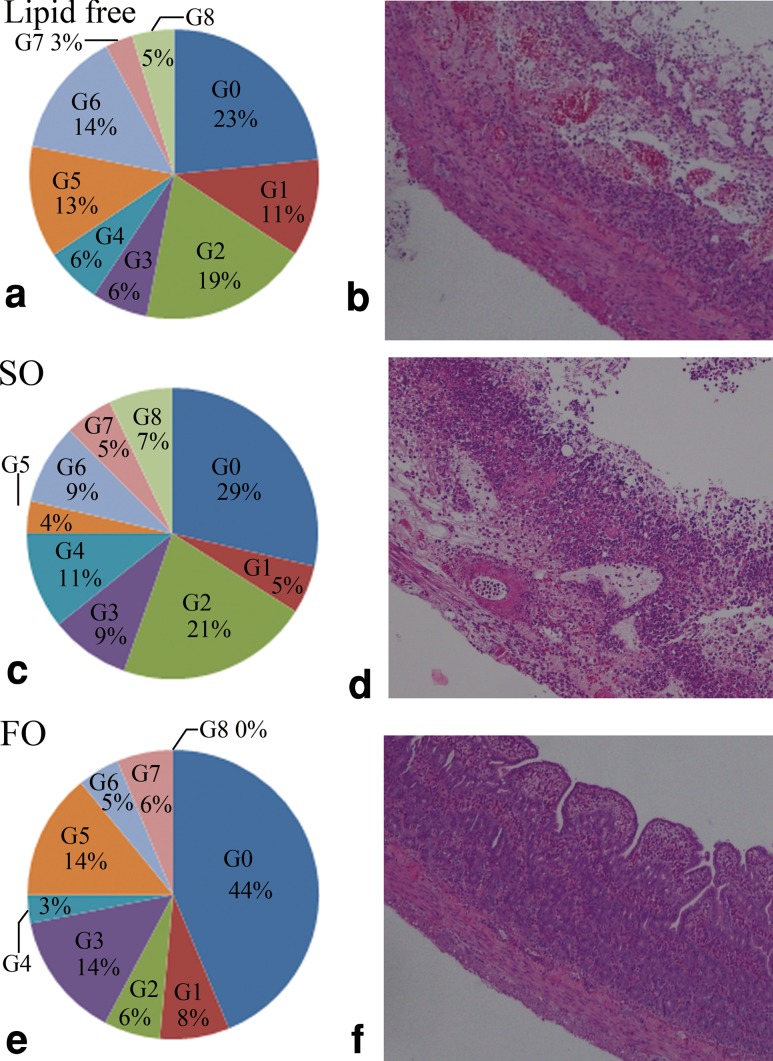
Representative findings of intestinal tissue specimens after ischemia−reperfusion injury (**b**, **d**, **f**) (hematoxylin−eosin staining×100). Tissue damage scores were also shown (**a**, **c**, **e**). The histological damage score was significantly lower in the fish oil group as compared with that in the soybean oil group (*P* = 0.0121)

There was no mucosal damage in an intestinal tissue after a sham surgery (Fig. 1). The intestinal tissue specimens from the rats in the soybean oil group exhibited severe mucosal epithelial necrosis and shedding, as well as deep-layer necrosis (Fig. 2d). The tissues from the rats in the fish oil group also showed deep-layer necrosis, but no mucosal epithelial necrosis. The rest of the damage in the fish oil group showed no or only mild intestinal tissue damage (Fig. 2f). The histological damage score was significantly lower in the fish oil group compared with that in the soybean oil group (*P* = 0.0121).

**Fig. 3:**
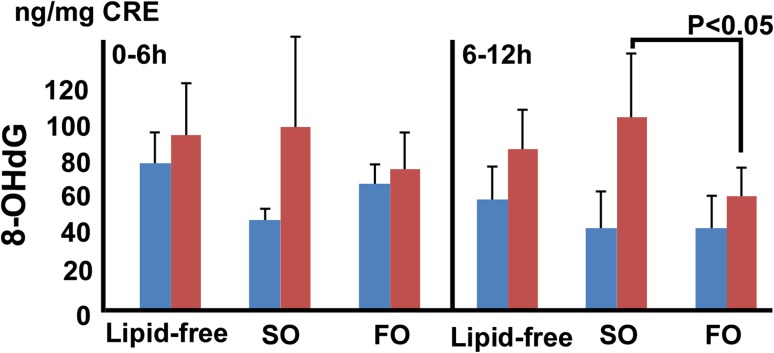
The 8-OHdG levels were significantly lower in the fish oil group than in the soybean oil group in the late phase (101.7 ± 38.1 vs. 61.7 ± 21.3 ng/mg CRE, respectively, *P* = 0.0267)

The 8-OHdG levels in the late-phase urine samples were significantly higher in the soybean oil group than in the fish oil group (101.7 ± 38.1 vs. 61.7 ± 21.3 ng/mg creatinine (CRE), respectively, *P* = 0.0267) (Fig. 3).

**Fig. 4:**
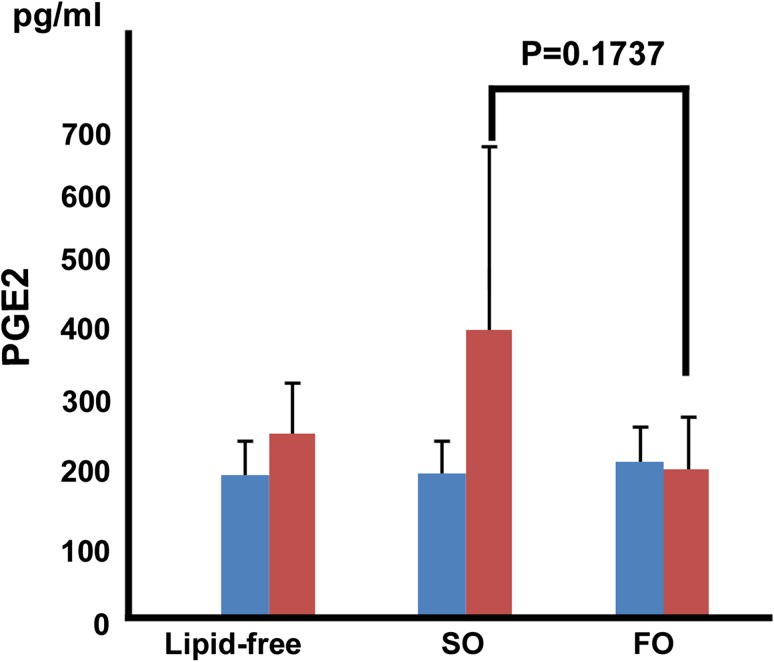
The PGE2 score at 6 h tended to be lower in the fish oil group than in the soybean oil group (214 ± 74 vs. 416 ± 258 pg/mL, *P* = 0.1737)

**Fig. 5:**
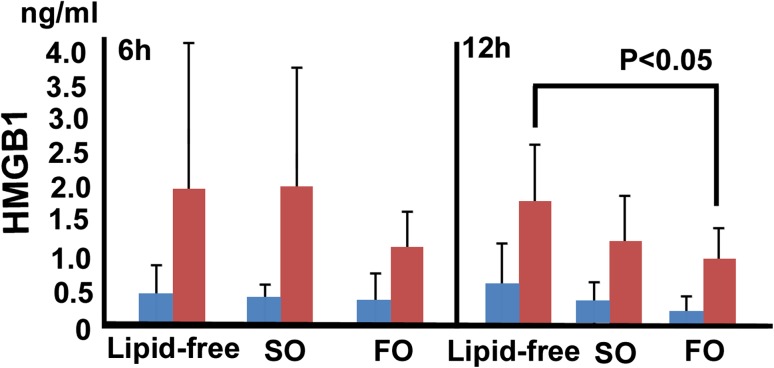
The plasma levels of HMGB1 were significantly lower in the fish oil group than in the lipid-free group (0.763 ± 0.32 vs. 1.4 ± 0.63 ng/ml, *P* = 0.0398)

The PGE2 score, based on the plasma levels of inflammatory eicosanoids, after 6 h of reperfusion tended to be lower in the fish oil group than in the soybean oil group (214 ± 74 vs. 416 ± 258 pg/mL, *P* = 0.1737) (Fig. 4). At 12 h after reperfusion, plasma levels of HMGB1, a mediator of endotoxic shock and sepsis, were significantly lower in the fish oil group (0.763 ± 0.32 ng/ml) than in the lipid-free group (1.4 ± 0.63 ng/ml, *P* = 0.0398) (Fig. 5).

## Discussion

Ischemia−reperfusion injury occurs frequently in small bowel transplantation, and often constitutes a major complication of this procedure.

**Fig. 6:**
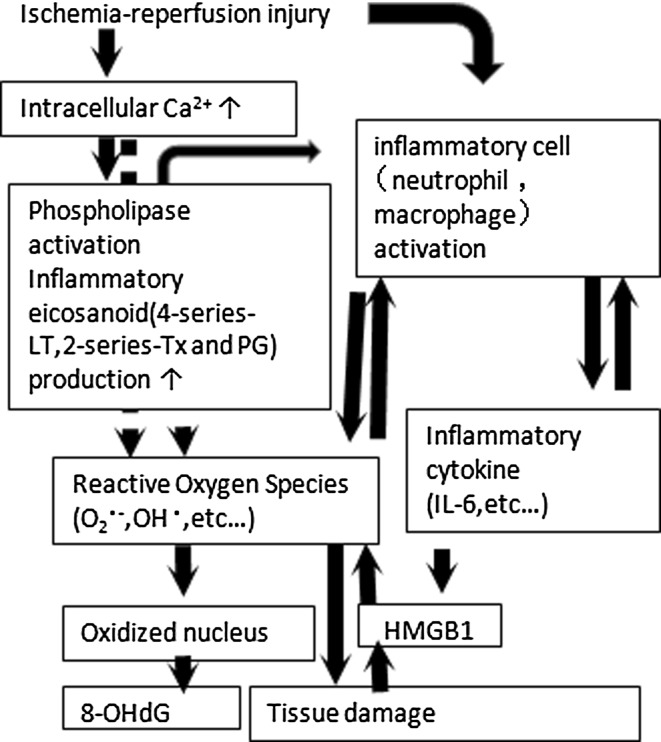
The mechanism underlying intestinal ischemia−reperfusion injury is typically described as a cascade

In the literature, the mechanism underlying intestinal ischemia−reperfusion injury is typically described as a cascade (Fig. 6). Ischemia−reperfusion injury causes an increase in intracellular calcium, which triggers phospholipase activation and up-regulation of inflammatory eicosanoids, such as the ‘4’ series of leukotrienes (LT), ‘2’ series of thromboxane (TX), and prostaglandin (PG). With the formation of reactive oxygen species (ROS), including superoxide and hydroxyl radicals, nuclear oxidation occurs, resulting in the formation of 8-OHdG as a metabolite. Urinary 8-OHdG formed by nuclear peroxidase appears in the urine in the late-phase of ischemia−reperfusion injury, particularly after 24 h [11, 12]. The production of ROS is associated with induction of cell membrane damage. In addition, neutrophils and macrophage are also activated, increasing the production of inflammatory cytokines. This process also leads to the production of HMGB1, which has been regarded as a mediator of late-phase inflammatory signaling in ischemic injury of organs such as the lung and liver [13, 14]. A characteristic finding of small-intestinal injury was that the plasma HMGB1 concentrations increased more rapidly as compared with that following injury to other organs [15, 16]. Lower plasma levels of HMGB1 were found in the fish oil group as early as at 6 h after the ischemia−reperfusion injury in the present study.

Fatty acids derived from fish oil, such as eicosapentaenoic acid (EPA), result in the formation of the ‘5’ series of PGs, TXs, and LTs. These substances are thought to down-regulate the inflammatory response [7]. In contrast, fatty acids derived from soybean oil, such as arachidonic acid (AA), result in the formation of the ‘2’ series of PGs and TXs and the ‘4’ series of LTs, which promote inflammatory responses [7]. Omega-3 lipid emulsions have been reported to down-regulate the production of inflammatory cytokines, such as IL-6 and TNFα [17]; however, only a limited amount of evidence has been accumulated. The present study was aimed at investigating the effect of fish-oil-derived fatty acids against ischemia−reperfusion injury of the intestine, as compared to other lipid components. The severity of the injury was assessed by evaluation of the changes in the plasma levels of inflammatory markers and oxidative stress markers, and the histopathologic tissue damage scores.

In the current study, fish oil administration significantly reduced the severity of histological damage in the fish oil group as compared with that in the soybean oil group. Reduction in the plasma levels of oxidative stress markers was observed, along with a decrease of the plasma HMGB1 levels. Therefore, the present observations indicate that the severity of tissue damage was reduced through down-regulation of oxidative stress and inflammatory responses. In addition, lower plasma levels of inflammatory eicosanoids observed in the present study also suggest that attenuation of the change in the omega-3/omega-6 ratio in the membranous lipid may play a major role in reducing the tissue damage. In the data, a few outliers made fairly large SD, especially in HMGB1 and PGE2. In addition to the delicate surgical animal model, dynamic changes of these parameters in vivo could result in these variabilities.

Prior administration of the omega-3 lipid emulsion reduced the plasma/urinary levels of inflammatory markers both in the early and late phases of ischemia−reperfusion injury. Consistent with the results of the current study, Byrne et al. [3] reported suppressed neutrophil adherence, which reduced the severity of ischemia−reperfusion injury, and also that omega-3 lipids mimic the early events in the injury. Furthermore, Sukhotnik et al. [2] reported decreases in the severity of intestinal mucosal injury and enterocyte apoptosis following intestinal ischemia−reperfusion injury in the rat.

These observations, including our own, suggest that the efficacy of fish oil may be attributable not only to a single step action in the late phase, but also to several steps in the inflammatory cascade. Therefore, administration of omega-3 lipids prior to intestinal ischemia may exert a significant beneficial effect against intestinal tissue injury.

Our current results indicate the clinical efficacy of omega-3 lipids in reducing intestinal ischemia−reperfusion injury commonly seen after intestinal transplantation. Future studies on omega-3 lipids are warranted for clarifying the mechanism of anti-inflammatory effect more precisely, and subsequently to establish the clinical efficacy of the lipids in a variety of critical conditions.
